# Human microglia show unique transcriptional changes in Alzheimer’s disease

**DOI:** 10.1038/s43587-023-00424-y

**Published:** 2023-05-29

**Authors:** Katherine E. Prater, Kevin J. Green, Sainath Mamde, Wei Sun, Alexandra Cochoit, Carole L. Smith, Kenneth L. Chiou, Laura Heath, Shannon E. Rose, Jesse Wiley, C. Dirk Keene, Ronald Y. Kwon, Noah Snyder-Mackler, Elizabeth E. Blue, Benjamin Logsdon, Jessica E. Young, Ali Shojaie, Gwenn A. Garden, Suman Jayadev

**Affiliations:** 1 Department of Neurology, University of Washington, Seattle, WA USA; 2 Biostatistics Program, Public Health Sciences Division, Fred Hutchinson Cancer Research Center, Seattle, WA USA; 3 Center for Evolution and Medicine, Arizona State University, Tempe, AZ USA; 4 School of Life Sciences, Center for Evolution and Medicine, Arizona State University, Tempe, AZ USA; 5 Sage Bionetworks, Seattle, WA USA; 6 Department of Laboratory Medicine and Pathology, University of Washington, Seattle, WA USA; 7 Institute for Stem Cell and Regenerative Medicine, University of Washington, Seattle, WA USA; 8 Department of Orthopaedics and Sports Medicine, University of Washington, Seattle, WA USA; 9 ASU-Banner Neurodegenerative Disease Research Center, Arizona State University, Tempe, AZ USA; 10 Division of Medical Genetics, University of Washington, Seattle, WA USA; 11 Brotman Baty Institute for Precision Medicine, Seattle, WA USA; 12 Cajal Neuroscience, Seattle, WA USA; 13 Department of Biostatistics, University of Washington, Seattle, WA USA; 14 Department of Neurology, University of North Carolina, Chapel Hill, NC USA

**Keywords:** Alzheimer's disease, Neuroscience, Gene regulatory networks, Transcriptomics, Ageing

## Abstract

Microglia, the innate immune cells of the brain, influence Alzheimer’s disease (AD) progression and are potential therapeutic targets. However, microglia exhibit diverse functions, the regulation of which is not fully understood, complicating therapeutics development. To better define the transcriptomic phenotypes and gene regulatory networks associated with AD, we enriched for microglia nuclei from 12 AD and 10 control human dorsolateral prefrontal cortices (7 males and 15 females, all aged >60 years) before single-nucleus RNA sequencing. Here we describe both established and previously unrecognized microglial molecular phenotypes, the inferred gene networks driving observed transcriptomic change, and apply trajectory analysis to reveal the putative relationships between microglial phenotypes. We identify microglial phenotypes more prevalent in AD cases compared with controls. Further, we describe the heterogeneity in microglia subclusters expressing homeostatic markers. Our study demonstrates that deep profiling of microglia in human AD brain can provide insight into microglial transcriptional changes associated with AD.

## Abstract (web summary)

Microglia, the innate immune cells of the brain, exhibit diverse functions and can influence Alzheimer’s disease progression. This study explores human microglial diversity in Alzheimer’s disease, uncovering unique disease-associated microglial populations and predicting underlying gene regulatory networks.

## Main

Alzheimer’s disease (AD) is pathologically characterized by extracellular amyloid-beta (Aβ) plaques, neuronal intracellular neurofibrillary tangles and neuroinflammation. Interest in neuroinflammation as a modifiable feature of AD pathology has grown in conjunction with genetic studies identifying AD risk variants localized to coding and non-coding regions of genes uniquely expressed by brain myeloid cells^1^. Microglia are resident innate immune myeloid cells of the brain and contribute to the neuroinflammatory processes hypothesized to promote AD pathophysiology^2–10^. Previous studies suggested that, in AD, microglia release inflammatory mediators that influence the behavior and function of surrounding neurons, and glia lose neuroprotective functions and initiate aberrant phagocytosis of synapses and neurons^3,7,11,12^. Microglia appear to contribute to tau spreading in model systems^13,14^ and are likely to be the primary cell type involved in Aβ removal, including the reduction in Aβ observed in response to antibody-based immunotherapy approaches^15^. As such, microglia inflammatory behaviors are relevant to therapeutic target design. However, large gaps remain in our understanding of microglia responses in AD brain.

Microglia phenotypes are differentiated by morphology, physiology and gene or protein expression patterns. Experiments performed in model systems suggest that these heterogenous features are likely to associate with specific functional microglia phenotypes^10,16–21^. Less is known about the heterogeneity of microglia phenotypes within the adult human brain, especially in the setting of specific disease states, such as AD. Single-cell and single-nucleus RNA sequencing (snRNA-seq) studies of fresh and frozen human cortical tissue have revealed multiple microglia transcriptional phenotypes in the context of AD and other brain pathologies^22–29^. Distinguishing transcriptomically distinct clusters enables the identification of candidate genetic and epigenetic factors regulating specific cellular behaviors, which might be leveraged in precision therapeutics approaches. However, standard snRNA-seq methods often include small numbers of microglia per individual. Low cell numbers may diminish the ability to map the full range of microglial transcriptional phenotypes and limit capacity to identify disease-associated gene expression change within a cluster or subcluster. We hypothesized that additional cellular processes and regulatory factors of microglia transcriptional phenotypes would be uncovered by using datasets that contain much larger numbers of microglia per individual sample.

We employed fluorescence-activated nuclei sorting (FANS) for PU.1 as a microglia enrichment technique for snRNA-seq. The myeloid marker PU.1 has been used to enhance the investigation of the epigenetics of microglia using assay for transposase-accessible chromatin using sequencing^30^, and, in the present study, it was applied to RNA sequencing (RNA-seq). This approach facilitated the acquisition of single-nucleus transcriptome profiles from thousands of microglia per subject. We generated microglia transcriptional profiles from a cohort of 22 individuals with and without AD, enabling us to annotate microglia clusters with plausible biological roles and identify differences in microglia between AD and control individuals. The large number of profiles in this dataset also allowed us to identify AD-specific subclusters within the microglia cluster typically annotated as ‘homeostatic’ based upon its gene expression profile^22,25,26,29^. In addition to homeostatic and inflammatory phenotypes described in previous reports, we uncovered microglial phenotypes with transcriptomic profiles that may give additional insight into AD pathogenesis. These findings provide new avenues for hypotheses testing in future studies on the roles of microglia in AD.

## Results

### FANS for PU.1 expression enriches microglia nuclei 20-fold

**Extended Data Fig. 1:**
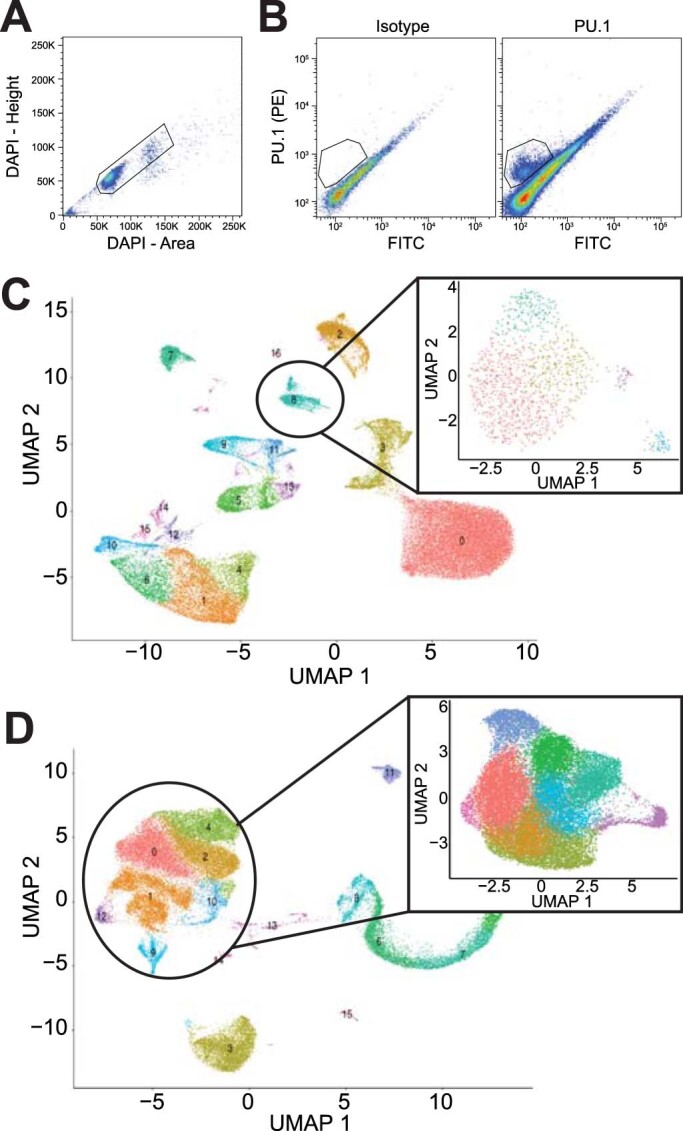
PU.1 enrichment increases the number of microglia nuclei and enhances microglia cluster resolution in snRNAseq studies. (**A)** Fluorescence-activated nuclei sorting plot of nuclei isolated from dorsolateral prefrontal cortex grey matter of postmortem human brain tissue demonstrate a DAPI-positive population from which later populations are drawn. (**B)** Isotype and PU.1 staining examples demonstrating the PU.1 positive population. (**C)** Unsorted snRNAseq data from four samples demonstrates multiple brain cell types and a small population of microglia (n = 1032 cells) that can be further subdivided into five clusters. (**D)** After PU.1 enrichment, a snRNAseq dataset from the same four individuals contains a larger number of microglia (n = 23,310 cells), and these microglia can be further discriminated into nine clusters.

We enriched nuclei isolated from postmortem human brain for microglia using FANS for expression of the myeloid-specific transcription factor PU.1 (Extended Data Fig. 1a,b). To confirm that PU.1 FANS was effective, we isolated and sequenced nuclei with and without PU.1 FANS (*n* = 4). We analyzed similar numbers of total nuclei in the unsorted (46,085; Extended Data Fig. 1c) and PU.1 sorted (41,488; Extended Data Fig. 1d) datasets. The PU.1 sorted dataset contained 20× more microglia nuclei defined by high expression of *C3*, *CD74*, *C1QB*, *CX3CR1* and *SPI1* (23,310 microglia nuclei) than the unsorted dataset (1,032 microglia nuclei). The microglia nuclei observed in the PU.1 sorted dataset also demonstrate further complexity as evidenced by more microglia clusters (Extended Data Fig. 1d).

**Fig. 1:**
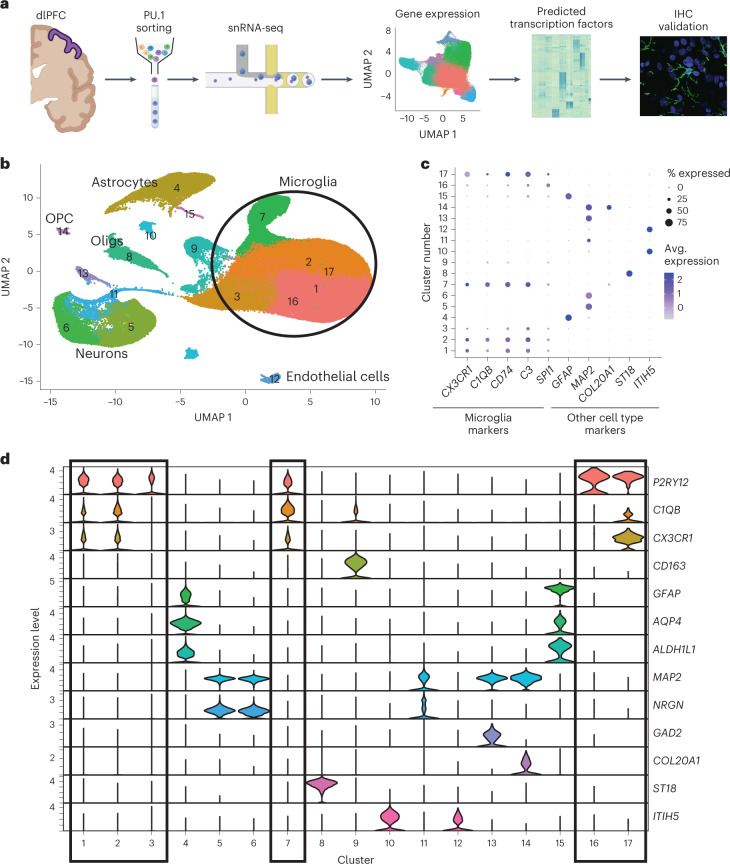
PU.1 enrichment yields a large dataset of microglia nuclei. **a**, Experimental design of 22 postmortem human dorsolateral prefrontal cortices (created in part with BioRender). **b**, UMAP of the PU.1 sorted nuclei from the 22-subject dataset demonstrates that, although other cell types, including neurons, astrocytes, oligodendrocytes (Oligs) and their progenitors (OPCs) as well as endothelial cells, are present, six clusters, including the three largest, are composed of microglia nuclei. **c**, Representative cell type marker genes (*x* axis) with the percent of nuclei that express a gene (size of dot) in each cluster (distributed along the *y* axis) and the average expression level (color intensity) are shown for microglia (*CX3CR1*, *C1QB*, *CD74* and *C3*), astrocytes (*GFAP*), neurons (*MAP2*), OPCs (*COL20A1*), Oligs (*ST18*) and endothelial cells (*ITIH5*) for each cluster. **d**, Gene expression of a wider set of cell type marker genes demonstrates that clusters 1, 2, 3, 7, 16 and 17 are composed of microglia. IHC, immunohistochemistry.

**Extended Data Fig. 2:**
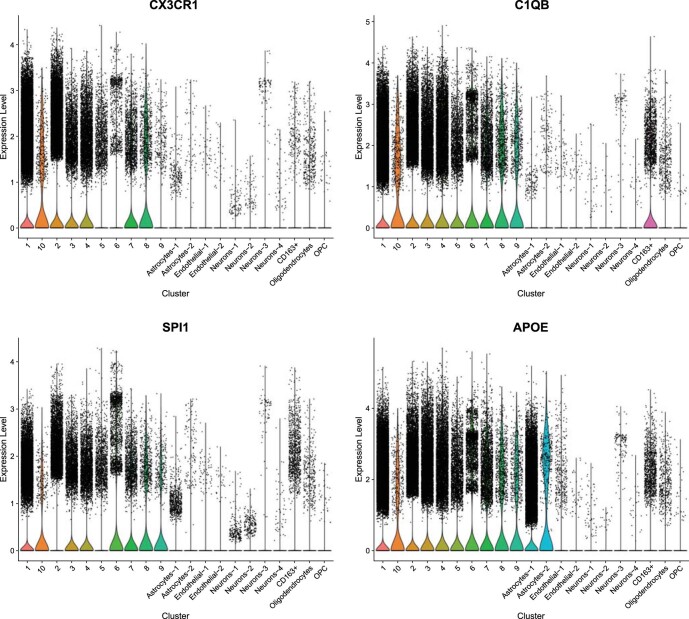
Gene expression of microglia marker genes in the 10 identified microglia subclusters. Microglia marker genes, *CX3CR1*, *C1Qb*, *SPI1* (PU.1), and *APOE* all demonstrate higher expression in the 10 microglia subclusters (numbered 1–10 on the left) than in other cell type clusters (labeled by cell type on the right).

**Extended Data Fig. 3:**
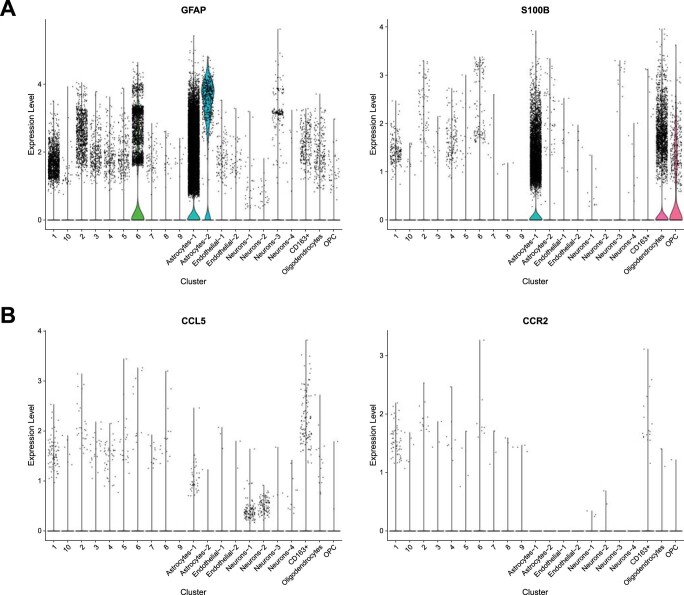
Gene expression of astrocyte and peripheral monocyte marker genes in the 10 identified microglia subclusters. (**A)** Astrocyte marker genes *GFAP* and *S100B* demonstrate higher expression in the two Astrocytes-1 and Astrocytes-2 subclusters than in the 10 defined microglia subclusters. While microglia cluster 6 does have expression of *GFAP*, it does not have expression of *S100B*. (**B)** Peripheral monocyte markers *CCL5* and have low expression in our dataset but are most highly expressed by the *CD163*+ cluster which was not included in our microglia dataset.

We next applied PU.1 FANS to a cohort of 22 individuals (Fig. 1a). After PU.1 FANS, samples retain a variety of non-myeloid cell types (Fig. 1b) while providing clear resolution of clusters demonstrating distinct microglia gene expression patterns (63% of the nuclei; Fig. 1c). The initial dataset consisted of 205,226 nuclei, with 200,948 nuclei (98%) passing quality control and doublet removal. Gene expression of cell type marker genes demonstrates that clusters identified as microglia (1, 2, 3, 7, 16 and 17; Fig. 1d) in the dataset have high expression of microglia markers and do not express canonical marker genes of other cell types. Thus, of the 200,948 nuclei, 127,371 were identified as microglia (Fig. 1d and Extended Data Fig. 2), with an average of 5,790 nuclei per individual. This dataset is the largest microglia per sample dataset generated thus far, including compared to other published datasets that have used alternative enrichment techniques. Detailed gene expression plots of both microglia (Extended Data Fig. 2) and astrocyte or peripheral monocyte markers (Extended Data Fig. 3) demonstrate high expression of microglia genes in the microglia subset dataset across all subclusters and the lack of other cell type and peripheral markers.

### Complexity of microglia states

**Fig. 2:**
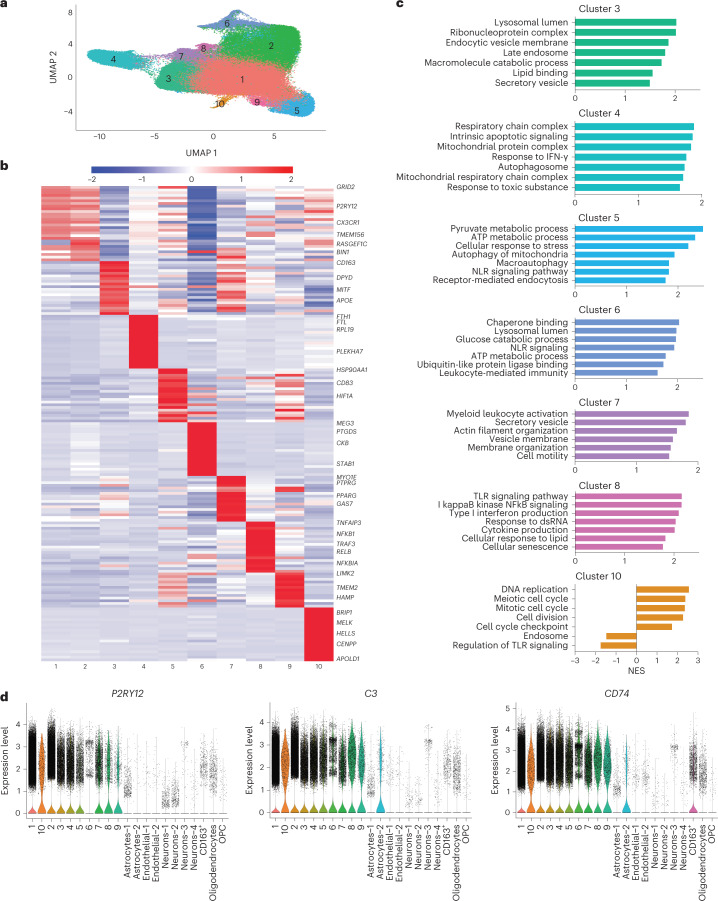
Microglia states have diverse gene expression and biological pathway correlates. **a**, UMAP of unbiased clustering on the nuclei from the six PU.1 sorted clusters (1, 2, 3, 7, 16 and 17, shown in Fig. 1d) meeting criteria for microglia from the 22-sample dataset contains 10 microglia clusters. **b**, Differential expression analysis comparing each cluster to all other clusters demonstrates distinct gene expression profiles for each. The top 25 genes from each cluster are displayed with gene names annotated on the right. Cluster 1 is high in expression of canonical microglia genes (*CX3CR1* and *P2RY12*). **c**, GSEA analysis of genes that differentiate each cluster from cluster 1 (‘homeostatic marker’) suggests distinct biological pathways. **d**, Canonical microglia marker gene expression in the microglia dataset versus other cell types sorted during PU.1 enrichment demonstrates enrichment of microglia marker gene expression in the 10 clusters. NES, normalized enrichment score.

Cluster analysis of the 127,371 nuclei with microglia-like expression identified 10 clusters (Fig. 2a) characterized by differentially expressed genes (DEGs) comparing the cluster to all other nuclei (Fig. 2b). Using gene set enrichment analysis (GSEA), we determined the enrichment of biological pathways in each cluster (Fig. 2c). There was little to no overlap in the DEGs defining each cluster or the biological pathways identified by GSEA, supporting the uniqueness of each cluster.

First, we found clusters with annotations similar to microglia phenotypes previously described in human brain. We identified cluster 1, the largest cluster, as the cluster enriched for homeostatic genes, including high expression of *CX3CR1* and *P2RY12* (refs. ^19,24–26^). We abbreviated this homeostatic-marker-expressing cluster as ‘HM’. HM was established as the basis for comparison to assess DEGs for other clusters, replicating the approach in previous publications^22,25,26^ (Supplementary Data 1). Cluster 4 was enriched for pathways involved in apoptosis, response to interferon-gamma (IFN-γ) and mitochondrial and respiratory functions (Fig. 2c), including Alzheimer, Parkinson and Huntington disease KEGG pathways. The most highly DEG in this cluster is *FTL*. Taken together, the profile of cluster 4 is suggestive of a degenerative or dystrophic phenotype^12,26^. Cluster 7 was characterized by expression of genes involved in migration and motility (Fig. 2b). Pathways enriched in cluster 7 included membrane organization and motility (Fig. 2c). Cluster 8 featured a canonical inflammatory phenotype with expression of classic inflammatory activation genes, including *NF*κ*B1*, *RELB* and *IL1*β (Fig. 2b)^2,31,32^. GSEA revealed that this cluster was enriched in NFκB signaling, interferon signaling, Toll-like receptor (TLR) signaling and RIG-I-mediated signaling pathways, indicating downstream effector inflammatory responses to stimuli (Fig. 2c). Additional Gene Ontology (GO) terms associated with cluster 8 included lipid synthesis and localization. Cluster 9 is defined by genes and pathways involved in senescence, iron homeostasis and cytokine production (Fig. 2b), including *CDKN1A*, *CEBPB*, *ZFP36* and *FTL*^33^. This profile is suggestive of senescent microglia^34^. Cluster 10 is defined by expression of genes involved in cell cycle regulation and DNA repair^35,36^ (Fig. 2b). The pathways enriched in cluster 10 confirm the relative increase of genes involved in cell cycle processes and a decrease of endosome and cytokine processing genes (Fig. 2c). As expected for microglia of varying activated and non-activated phenotypes, genes such as *P2RY12* varied in expression, whereas microglia genes, such as *C3* and *CD74*, had more similar representation across the microglia subclusters (Fig. 2d).

**Extended Data Fig. 4:**
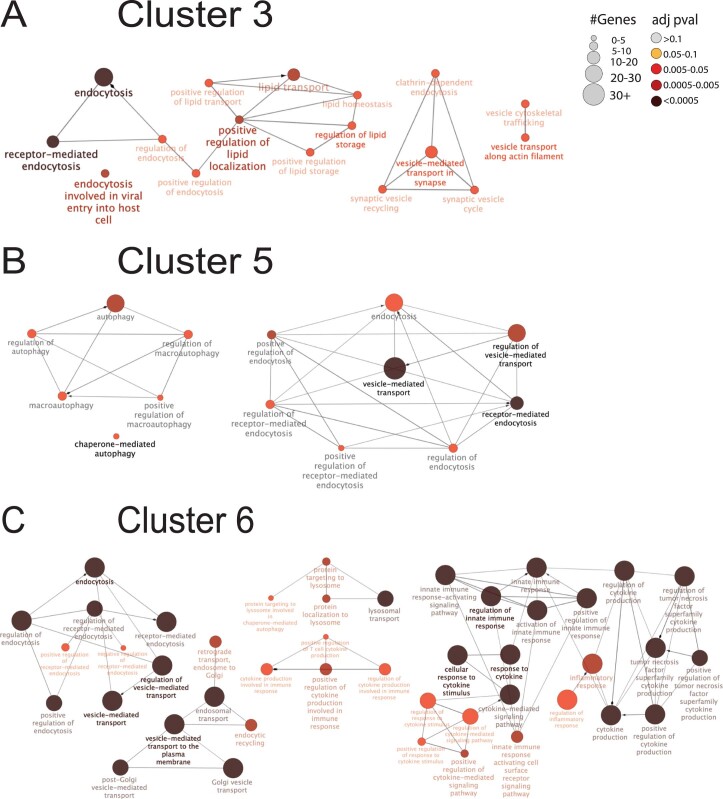
Gene Ontology Network Enrichment of the Endolysosomal Microglia Clusters 3, 5, and 6. The genes differentially expressed in Clusters 3, 5, and 6 were employed to drive a network-based gene ontology enrichment using the Cytoscape application ClueGO. This approach uses a one-sided positive enrichment algorithm that employs hypergeometric testing, with a kappa-threshold of 0.4 to optimize the biological process connections. Each term drawn into the network was initially filtered for multiple testing corrections threshold of *p* < 0.05, and hierarchically weighted for terms with a Benjamini-Hochberg correction value of *p* < 0.01. The nodes are represented within each network based upon two factors: number of genes (size of circle) and statistical significance (bright red = Benjamini-Hochberg corrected *p* < 0.05; dark brown = Benjamini-Hochberg corrected *p* < 0.0005). **(A)** In Cluster 3, three small subclusters were identified associated with endocytosis, receptor mediated endocytosis, and lipid binding and synthesis (far left); a second subcluster centers on synaptic endocytosis (middle group); and a third involves vesicle transport (far right). **(B)** In Cluster 5, a subcluster of terms was identified spanning autophagy, receptor mediated autophagy, and the regulation of these two terms (far left); a second larger cluster identifies endocytosis and receptor-mediated endocytosis, processes linked with autophagic regulation, as well as vesicular transport. **(C)** In Cluster 6, three subnetworks were identified within the ELN space, demonstrating active endocytosis, lysosomal processes and transfer between these compartments and the trans-golgi network (left). A second large cluster of terms was identified associated with innate immune function, activation, and regulation, as well as linked inflammatory processes (right side).

Next, we found three clusters, clusters 3, 5 and 6, not previously described in human brain. These clusters were distinguished by their endolysosomal network (ELN) gene expression and enrichment for ELN pathway signatures relative to HM microglia. We, therefore, annotate them collectively as ELN. Cluster 3 is defined by genes implicated in aggregate protein internalization (Fig. 2b)^19,20,24–26,37^ phagocytosis and vesicle-mediated transport^32^. Pathways enriched in cluster 3 include endosome and lysosome pathways as well as catabolism and lipid binding but no inflammatory processes (Fig. 2c). Genes involved in glycolysis have lower expression in cluster 3, differentiating it from the two other ELN clusters, suggesting that these cells have not undergone the metabolic switch to glycolysis observed in the microglia inflammatory phenotype^38^. Clusters 5 and 6 displayed an ELN signature, although they also appeared metabolically active with distinct inflammatory characteristics. Cluster 5 had increased expression of *HSP90AA1*, *HIF1A* and *BAG3* in addition to other heat shock protein genes (Fig. 2b), suggesting that these cells are responding to external stress. Genes driving glycolysis were also higher in cluster 5 when compared to HM, possibly reflecting a switch to glycolysis in these cells^38^. The pathways enriched in this cluster indicate that it is active in endocytosis, autophagy and mitophagy (Fig. 2c). Cluster 6 is characterized by metabolic activity genes and stress response pathways similar to cluster 5 (Fig. 2c) although with an additional component of interferon signaling suggested by significantly higher levels of *IRF3*, *IRF5* and *IRF7*. Cluster 6 also showed increased expression of cytosolic DNA/RNA recognition and antiviral genes, including *IFIT2*, *IFIT3* and *TRIM22* (ref. ^39^), as well as the pattern recognition receptor *CARD9* and mediators of the NLRP3 inflammasome^31,32,40–43^. Of note, unique to cluster 6, we found that the DNA repair genes *ATM* and *RNASEH2B* were lower in expression. Whereas *IL1*β was increased in cluster 6 compared to HM, even higher levels of *IL1*β and expression of other inflammatory effector molecules, such as *NFkB1*, were found in cluster 8, the more canonical ‘inflammatory’ cluster. Pathways enriched in cluster 6 also support upstream inflammatory responses to danger-associated molecular pattern stimuli, such as enrichment in NOD-like receptor (NLR) signaling. In addition, we used alternative methodology to identify biological pathways and the links between them, providing validation for the ELN functions of clusters 3, 5 and 6 (Extended Data Fig. 4). Supplementary tables containing the genes driving the presence of each node in the network are available on Synapse.

**Extended Data Fig. 5:**
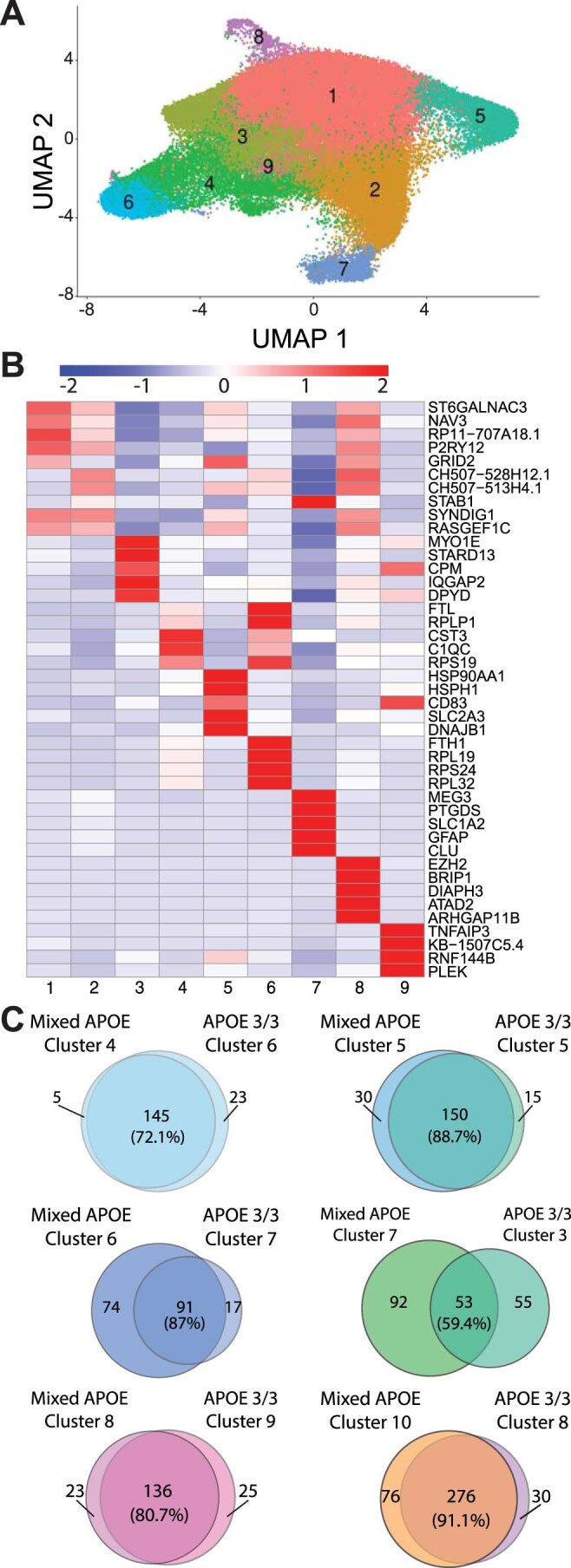
*APOE* ε3/ε3 genotype does not substantially alter microglial clustering in human autopsy brain. (**A)** UMAP of unbiased clustering on 13 samples of only *APOE* ε3/ε3 individuals shows 9 clusters. (**B)** Similar to the clusters identified in the Mixed *APOE* genotype dataset, the clusters identified in the *APOE* ε3/ε3 genotype dataset are distinct by gene expression. The top 5 genes are displayed for each cluster. (**C)** Venn diagrams demonstrating overlap between clusters from the Mixed *APOE* and *APOE* ε3/ε3 cohorts demonstrating significant overlap in gene expression profiles.

Despite the role of *APOE* in risk and progression of AD^44^, to date no studies have defined microglia states in individuals with a specific *APOE* genotype. Because most (13/22) of our samples were homozygous for the *APOE* ε3 allele, we generated a subset of our dataset that consisted entirely of *APOE* ε3/ε3 individuals (seven controls and six AD pathology, nine females and four males; 75,018 microglia nuclei). After re-normalizing and re-clustering, we identified nine clusters of microglia (Extended Data Fig. 5a). These clusters were defined by genes similar to those that defined the clusters in the Mixed *APOE* genotype dataset (Extended Data Fig. 5b and Supplementary Data 2). We found that, in most clusters, the DEGs were very similar (~60% or higher match) to those in the Mixed *APOE* dataset (Extended Data Fig. 5c). The HM, neurodegenerative, inflammatory, cell cycle and endolysosomal clusters were similar to those of the Mixed *APOE* cohort. This suggests that the presence of multiple distinct ELN microglia clusters is common in the human brain even when controlling for *APOE* genotype.

### Microglia cluster-specific transcription factor regulatory networks

**Fig. 3:**
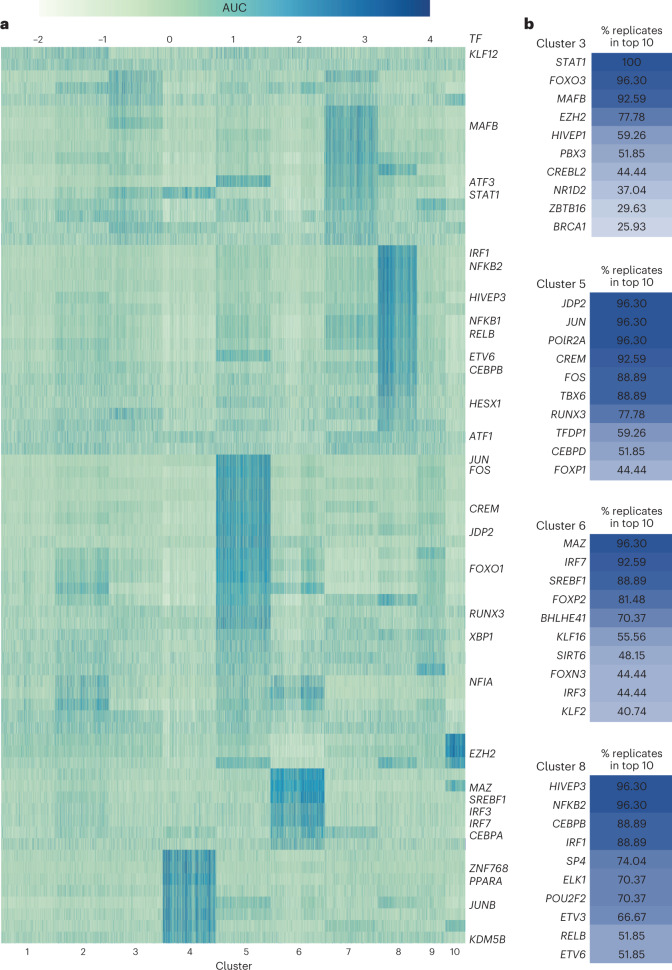
Transcription factor regulatory networks are specific to microglia phenotypes. **a**, SCENIC workflow identified transcription factor regulated networks associated with different phenotypic clusters of microglia. Heat map of area under the curve (AUC) of the transcription factor (regulon activity in clusters). **b**, Percentage of instances where transcription factor regulons occured in the top 10 regulon specificity scores per cluster (out of 27 permutations). Darker color indicates higher percentage of replication.

**Extended Data Fig. 6:**
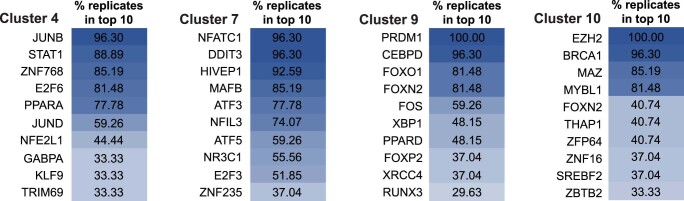
Top transcription factors for each cluster from the full cohort microglia subclusters are unique and represent biological function switches. These are the transcription factors that most often drove gene expression in clusters 4, 7, 9, and 10. Values denote the percentage of replicates of permutations of the dataset where that transcription factor was unique to the given cluster, with darker color indicating higher percentages. Note that while a few similar transcription factors are seen in multiple clusters, particularly the most prevalent transcription factors are unique, and are representative of gene expression driving biological functions in these clusters.

**Extended Data Fig. 7:**
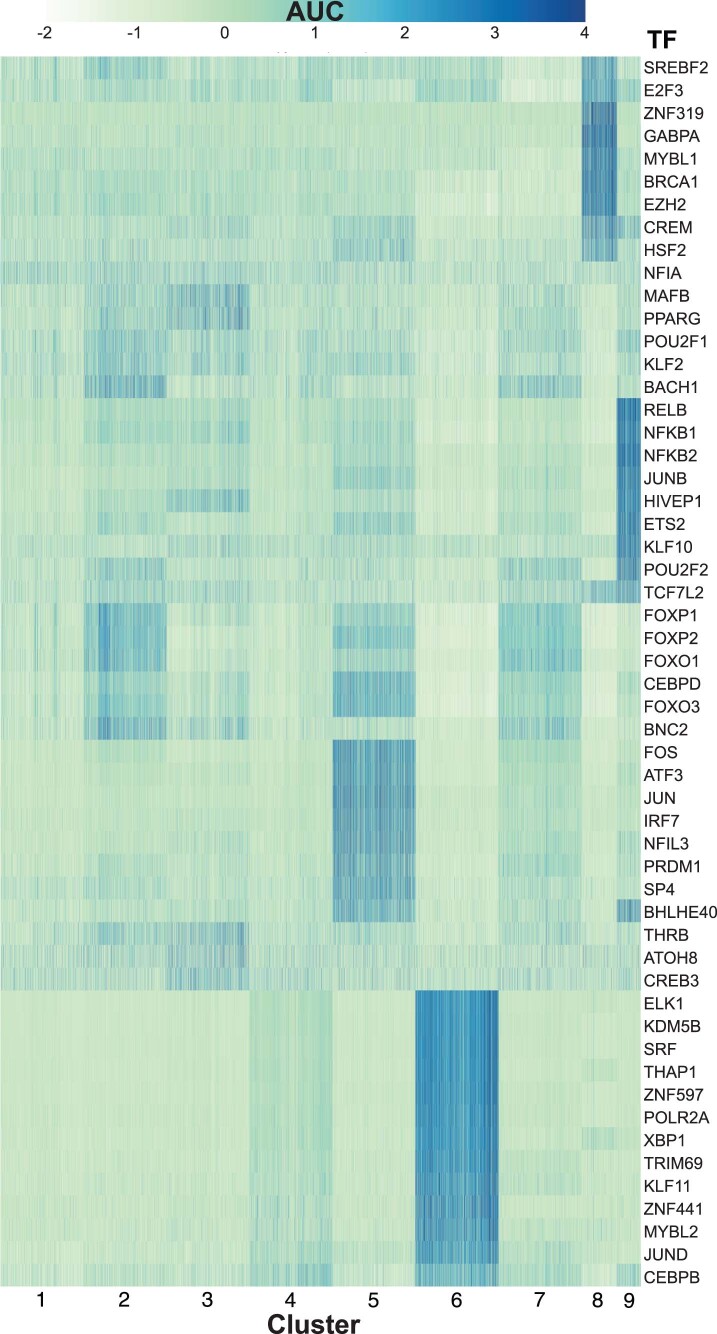
Transcription factor regulatory networks are specific and unique to subpopulations of microglia within the *APOE* ε3/ε3 genotype clusters. Similar to the larger dataset, transcription factors driving gene expression within clusters are distinct when the data is comprised of only *APOE* ε3/ε3 allele carriers.

To characterize the regulatory networks of the populations in the dataset, we identified the top transcription-factor-driven networks (regulons) controlling gene expression in each of the microglia clusters (Fig. 3). Each cluster is defined by a specific set of regulons (Fig. 3a), supporting the hypothesis that the differential gene expression characterizing each cluster is determined by transcriptional regulation mechanisms. To demonstrate the diversity of regulons predicted to drive gene expression in different clusters, we highlight cluster 3, cluster 5, cluster 6 and cluster 8 (Fig. 3b). Each of these clusters shows a different set of regulons that appear as one of the top 10 for that cluster repeatedly across permutations of the analysis. For example, cluster 5 shares a glycolytic and endolysosomal phenotype with cluster 6 but does not share the interferon response factor regulons predicted for cluster 6. In addition, whereas we observed *IRF1* and *NFKB2* regulons in cluster 8, the canonical ‘inflammatory’ effector cluster, we observed additional (and different) interferon response factor regulons in cluster 6. The top regulons for other clusters also differ from each other (Extended Data Fig. 6). *MAFB*, a transcription factor associated with anti-inflammatory gene regulation, was top of the list in cluster 3 (ref. ^16^). In contrast, regulons directed by transcription factors typically associated with antiviral responses, *IRF7*, and to a lesser extent *IRF3*, were top of the list in cluster 6, consistent with the observation that these cells are also enriched for nucleic acid recognition and endolysosomal pathways (Fig. 3b). The top regulons for the *APOE* ε3/ε3 subset of individuals demonstrate similar unique diversity to those described in the larger dataset, again suggesting homology across *APOE* genotypes (Extended Data Fig. 7). Together, these inferred gene networks and their transcription factor regulons demonstrate the diversity of the clusters identified here and provide potential regulatory targets for future studies to investigate.

### Microglia transcriptomic progression takes multiple paths

**Fig. 4:**
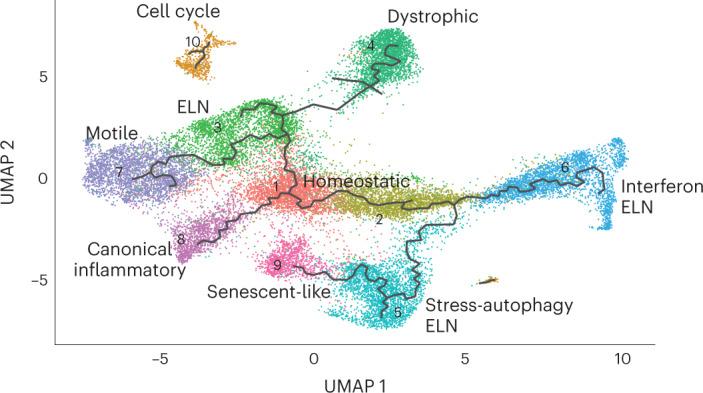
Microglia transcriptomic progression may take multiple paths. Monocle trajectory inference applied to the microglia dataset demonstrates that multiple phenotypic options radiate outward from cluster 1. Each branch has several potential endpoints, suggesting that microglia may not progress along a single staged linear trajectory but, instead, proceed through one of several transition states to reach various transcriptomic end phenotypes.

Experiments in model systems with defined stimuli have demonstrated the potential of microglia to acquire diverse phenotypes. However, understanding the progression and phenotypic switches acquired by human microglia in vivo is challenging. We employed our single-nucleus dataset to investigate microglia transcriptomic transitions using the Monocle3 (ref. ^45^) trajectory inference method (Fig. 4). We asked which cluster may be end state versus transition state as a hypothesis-generating exercise. The resulting branching trajectories suggest that multiple transition states radiate out from HM, the cluster enriched for homeostatic gene expression, supporting the hypothesis that ‘homeostatic’ microglia may transition to multiple endpoint phenotypes in humans as predicted by model studies^10,16,17^. We found relationships between clusters that were not immediately apparent when exploring DEGs and GSEA alone. Trajectory analysis revealed a branch point where cell progression continues to either the autophagic stress ELN cluster (cluster 5) or the inflammatory ELN, cluster 6 (Fig. 4). Cluster 5 is adjacent to the senescent-like cluster (cluster 9), consistent with the notion that autophagy and senescence are related biological pathways and endpoints. Similar to work by Nguyen et al.^26^, the motile cluster (cluster 7) is another endpoint.

### Inflammatory ELN profile microglia are increased in AD cases

**Fig. 5:**
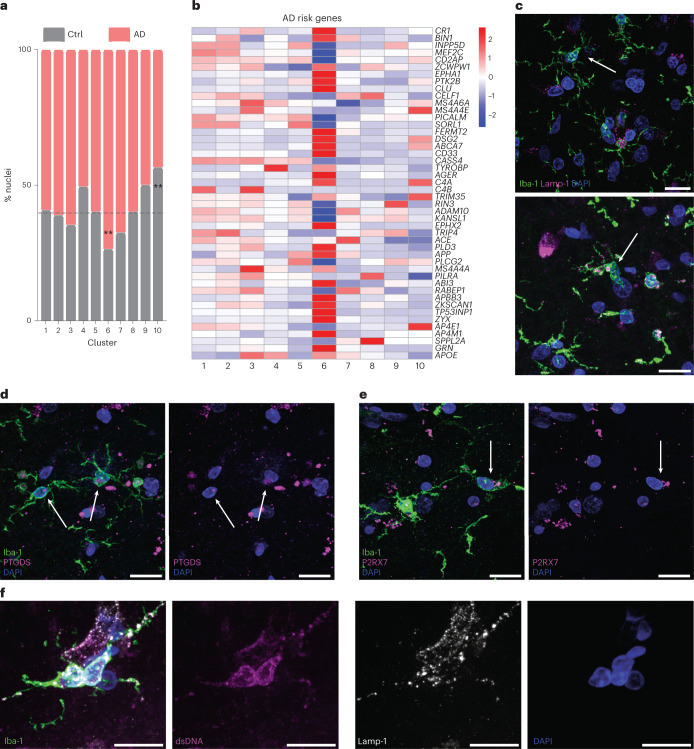
Cluster 6 demonstrates enrichment of AD risk genes and suggests the presence of dysregulated lysosomal and cytosolic DNA regulation in microglia in AD. **a**, Cluster 6 is significantly increased in AD brain (chi-square FDR-corrected *P* = 0.0064), whereas cluster 10 is increased in control (Ctrl) samples (chi-square FDR-corrected *P* = 0.0006). **b**, Heat map of AD-associated risk gene expression across microglia clusters shows stronger differential expression in cluster 6. **c**, Demonstration of lysosome morphology heterogeneity in microglia. Representative images from an AD case demonstrate microglia (Iba-1, green) with heterogeneity in morphology and Lamp-1 signal. Examples are of a ramified (top arrow) and greater lysosome (Lamp-1, magenta) signal and an ‘activated’ or less-ramified phenotype (bottom arrow). **d**, Representative microglia (Iba-1, green) with high PTGDS (red) expression, a cluster 6 marker in an AD case. **e**, Representative microglia (Iba-1, green) with high expression of P2RX7 (magenta) expression, a cluster 6 marker in an AD case. **f**, A representative example of activated microglia with both large numbers of lysosomes (Lamp-1, white) and cytosolic dsDNA (magenta) in an AD case. All representative images in **c**–**f** display staining replicated in multiple fields and at least three human brains. All scale bars represent 15 µm. **Corrected *P* < 0.01.

**Extended Data Fig. 8:**
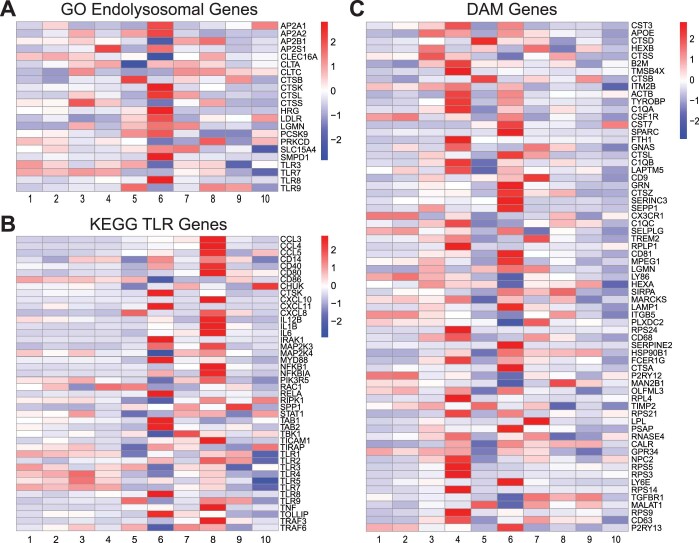
Gene sets demonstrate differential expression by cluster based on predicted biological function. Heatmaps of: (**A)** the Gene Ontology (GO) set of Endolysosomal genes demonstrate significant upregulation in Cluster 6. (**B)** The Kyoto Encyclopedia of Genes and Genomes (KEGG) set of Toll-Like Receptor (TLR) genes demonstrates significant upregulation in Cluster 8 as would be expected given the classical inflammatory phenotype observed in the gene expression. (**C)** The ‘Disease Associated Microglia’ (DAM) gene list from Keren-Shaul et al. 2017 shows upregulation of genes across multiple clusters in our human dataset. This replicates other human studies which have not identified a single DAM cluster unlike studies involving mouse models of Alzheimer’s disease.

Both homeostatic-marker-expressing and canonical inflammatory clusters (HM and cluster 8, respectively) were equally represented by both AD and control brain. In contrast, we found that cluster 6 had more AD nuclei than would be expected in our dataset (adjusted *P* = 0.006), suggesting that AD-relevant processes may be represented in the profile of this cluster (Fig. 5a). Multiple AD genome-wide association studies have identified risk alleles associated with genes expressed in microglia or myeloid cells^4^. Using a list of 46 genes in single-nucleotide polymorphism loci associated with altered AD risk^44,46^, we used GSEA to assess enrichment of these genes in the 10 identified microglia clusters. We observed that more AD risk genes are differentially expressed in cluster 6 (Fig. 5b) compared to all other clusters (adjusted *P* < 0.001). Gene expression of *PICALM*, *SORL1* and *PLCG2* were significantly lower in cluster 6 compared to the rest of the clusters, whereas other genes, including *APP*, *APOE* and *BIN1*, were significantly higher in expression (all adjusted *P* < 0.001; Fig. 5b). Using an alternative set of ELN genes, we demonstrate significant differential expression in cluster 6, whereas TLR-associated genes were more often highly expressed in cluster 8 (Extended Data Fig. 8a,b). Genes associated with ‘disease-associated microglia’ were enriched across multiple clusters, in contrast to a single cluster observed in AD mouse models^19^ (Extended Data Fig. 8c). We further investigated whether a microglia phenotype present in healthy aging was reduced in AD brain. We determined that cluster 10, the cluster differentially expressing cell cycle regulatory genes, is larger in control brain compared to AD brain (Mixed *APOE* adjusted *P* < 0.001 and *APOE* ε3/ε3 adjusted *P* < 0.001; Fig. 5a). Cluster 10 is the smallest cluster in our dataset and, therefore, deserves replication; however, these data suggest that AD pathology may involve a detectable reduction in a microglia cluster enriched for cell cycle and DNA repair genes.

### Protein expression of microglial phenotype markers

**Extended Data Fig. 9:**
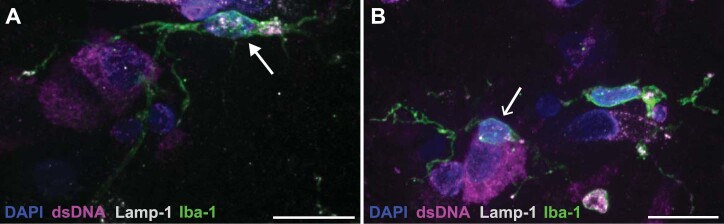
A subset of microglia demonstrate dsDNA signal and greater Lamp-1 signal in AD brain. (**A)** A representative example of staining observed in multiple fields across at least three humans shows an activated microglia with both large numbers of lysosomes (Lamp-1, white), and cytosolic dsDNA (magenta) in an AD case (filled arrowhead). (**B)** Microglia (pointed arrowhead) without cytosolic dsDNA immunoreactivity (magenta) in the same case and tissue section appear ramified with less Lamp-1 signal. All scale bars represent 15 microns.

The transcriptomic data predict heterogeneity in microglia endolysosomal phenotypes in both aged control and AD brain. Immunostaining for LAMP1, a lysosomal marker, revealed a spectrum of lysosomal phenotypes with varying lysosomal size and number (Fig. 5c). Microglia expressing cluster 6 markers were identified in AD brain by immunolabeling for the protein products of *PTGDS* (Fig. 5d) and *P2RX7* (Fig. 5e), both highly expressed genes in that subcluster. Cluster 6 and cluster 8 microglia both demonstrate differential expression of genes involved in the detection of DNA/RNA molecules, suggesting that microglia may be activated by exposure to cytosolic nucleic acids. To assess the presence of cytosolic nucleic acids in microglia, we immunolabeled microglia for double-stranded DNA (dsDNA) (Fig. 5d). We observed that microglia with immunoreactivity for dsDNA also contained enlarged lysosomes, whereas other microglia in the same tissue section had normal lysosome size and no immunoreactivity for dsDNA (Extended Data Fig. 9). These findings suggest that microglia heterogeneity reflected by gene expression patterns may represent morphological phenotypes that can be detected in human tissue.

### An AD-specific state exists within the cluster enriched for homeostatic genes

**Fig. 6:**
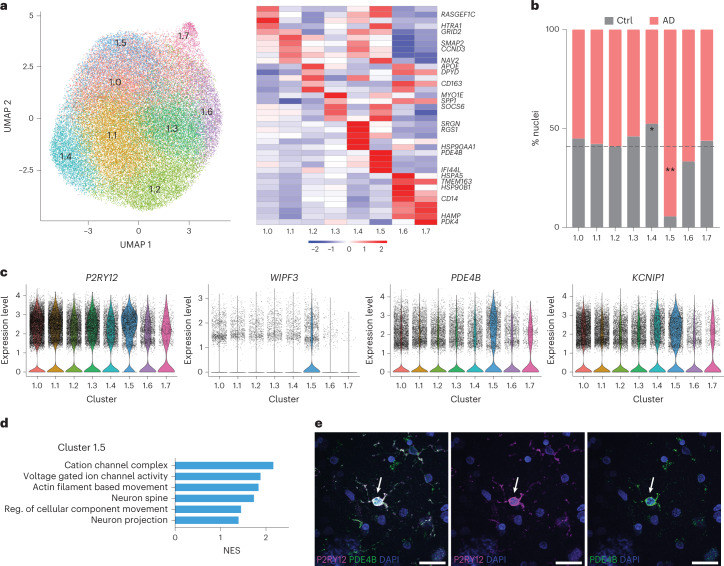
Within microglia expressing homeostatic markers there is a subcluster uniquely enriched in AD brain samples. **a**, Subclustering of cluster 1 homeostatic marker microglia (HM) revealed seven subpopulations defined by differential gene expression. **b**, Subcluster 1.5 is greatly expanded in AD brain samples (chi-square FDR-corrected *P* = 6.2936 × 10^−13^), whereas subcluster 1.4 is increased in control (Ctrl) brain samples (chi-square FDR-corrected *P* = 0.0194). **c**, Gene expression of *P2RY12* demonstrates high expression across the HM subclusters, with highest expression in subcluster 1.5. Genes differentially expressed in subcluster 1.5, such as *WIPF3*, *PDE4B* and *KCNIP*, also demonstrate high expression. **d**, Pathway enrichment in subcluster 1.5 demonstrates unique enrichment for motility, ion channel activity and neuron-related processes. **e**, Immunohistochemistry validates the presence of double-positive high P2RY12-expressing and PDE4B-expressing microglia in AD brain. Representative images display staining replicated in multiple fields and at least three human brains. Scale bars, 25 µm. *Corrected *P* < 0.05; **corrected *P* < 0.01. Reg., regulation.

**Extended Data Fig. 10:**
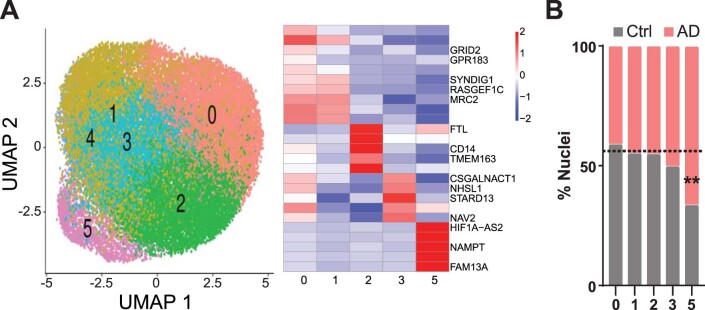
The *APOE* ε3/ε3 genotype cohort also demonstrates a subcluster of the homeostatic-marker-expressing cluster increased in AD brain. (**A)** The results from the full dataset were replicated in the pure *APOE* ε3/ε3 allele cohort, suggesting multiple subpopulations exist within the ‘homeostatic’ cluster. These subpopulations are distinct by gene expression despite being comprised of one ‘homeostatic’ subpopulation. (**B)** Within the *APOE* ε3/ε3 cohort ‘homeostatic’ Cluster 1, there is a Cluster 1.5 that is significantly increased in AD brain like we found in the full cohort (Fig. 6; Chi-squared FDR corrected *p* = 6.7673 × 10^−6^). **= *p* < 0.01.

We reasoned that, given the known inflammaging changes associated with human brain molecular profiles, it could be challenging to detect AD-specific signatures when studying polarized subpopulations in AD and aged brain tissue. We turned our attention to the complexity within HM. As previously reported, we observed that HM is the largest population and proportionally similar in AD and aged control brain samples^22,25,26^. This population has not been extensively characterized to understand the impact of AD on gene expression within homeostatic microglia. Our dataset studied over 50,000 microglia enriched for homeostatic markers, enabling detection of subclusters within the HM microglia population. Subclustering HM microglia revealed seven populations with differential gene expression (Fig. 6a). We found that, in contrast to the larger microglia dataset, there was a subcluster nearly unique to AD cases. This AD-specific microglia subcluster, subcluster 1.5 (Fig. 6b), was almost exclusively composed of AD microglia, suggesting that it may be uniquely driven by AD pathology (adjusted *P* < 0.001). In contrast, subcluster 1.4 was overrepresented by control nuclei (Fig. 6b; adjusted *P* < 0.05). HM subclusters showed high expression of *P2RY12*, with the highest expression in subcluster 1.5 (Fig. 6c). Other gene expression markers of HM subcluster 1.5 were more specific, including the DEGs *WIPF3*, *PDE4B* and *KCNIP* (Fig. 6c). To begin to characterize the putative biological processes represented in these subclusters, we performed GSEA as above. Subcluster 1.5 had a unique profile of enrichment for genes involved in cellular motility and calcium signaling (Fig. 6d). Using immunohistochemistry, we validated the presence of double-positive high P2RY12 and PDE4B protein expression in microglia in human AD brain (Fig. 6e). We additionally verified these results in our cohort of all *APOE* ε3/ε3 individuals. We confirmed that, again, the microglia cluster enriched for homeostatic gene expression could be divided into multiple populations with distinct gene expression and that one such cluster was enriched in AD cases (Extended Data Fig. 10).

## Discussion

This study identified 10 distinct microglia clusters from aged human brain. These included previously described homeostatic, senescent and inflammatory microglia transcriptional phenotypes as well as additional clusters of transcriptional specification, which may give insight into AD pathogenesis, providing a platform for hypothesis generation. We describe the diversity of microglia clusters with endolysosomal gene signatures, one of which is enriched with nucleic acid recognition and interferon regulation genes. Inferred gene networks predict that individual clusters are driven by distinct transcription factors, lending further support for the functional diversity of clusters. Using trajectory inference analysis, we observed transitions in microglia phenotypes and predicted relationships that can be tested experimentally. AD cases were distinguished by the emergence of a subcluster expressing homeostatic genes (subcluster 1.5) that was characterized by altered transcription of genes involved in calcium activation, response to injury and motility pathways.

Endolysosomal function is critical for trafficking and degrading pathologic proteins, and dysfunction of the ELN is implicated in AD pathogenesis. Less is known about the implications of ELN dysfunction specifically in AD microglia^47^. This study identified three microglia clusters, clusters 3, 5 and 6, distinguished by expression of ELN components, each with a distinct pattern of endocytosis, vesicle trafficking, endolysosomal and autophagosome pathway gene expression. Impaired microglial endolysosomal function is proposed to contribute to AD pathogenesis through insufficient amyloid clearance^1,47^. However, the ELN in myeloid cells also plays a critical role in identifying and processing foreign microbes, including initiation of TLR and interferon signaling^31,48^. Gene expression in cluster 6 (the subtype overrepresented in AD cases) was characterized by enrichment for expression of genes involved in lysosomal and vesicular function and concomitantly increased expression of interferon regulatory factor and inflammasome activation genes^40,42,49,50^. Although there is not a single ‘DAM’ phenotype in human AD, we did observe a type 1 interferon response cluster distinct from a dystrophic or canonical inflammatory phenotype^18,19,37,51^. We hypothesize that *IRF* expression here may reflect exposure to danger-associated molecular pattern molecules, including nucleic acids from microglia or neighboring cells. This hypothesis is supported by our observation of microglia with immunoreactivity to cytosolic dsDNA and amoeboid morphology (Fig. 5). These findings align with murine and in vitro studies demonstrating that amyloid fibrils contain nucleic acids and induce a type I interferon response in microglia, leading to synapse loss^50^. Song et al.^49,52^ reported a key role for the ELN in nucleic acid degradation and the interferon response to DNA damage after release of nuclear DNA into the cytosol, consistent with the phenotype that we describe here. Cluster 6 microglia not only were increased as a population in AD cases but also demonstrate significant differential expression of a number of genes associated with genetic risk for AD (Fig. 5). This observation supports the hypothesis that inflammatory cell dysfunction is an important contributor to AD risk and suggests that the subtype of microglia represented by cluster 6 may be a microglia subtype to target for therapeutic intervention.

We leveraged our dataset to apply trajectory methods to infer the potential relationships between microglial transcriptional phenotypes. Dystrophic (cluster 4) and senescent (cluster 9) clusters emerge as ‘end states’, demonstrating how computational trajectory inference can map microglial phenotypes consistent with predictions from previous empirical data^10,16,17^. Nevertheless, as with all bioinformatic analyses, these results should be viewed as hypothesis generating. Trajectory analysis also showed that the autophagic stress and inflammatory ELN clusters (cluster 5 and cluster 6, respectively) represented a branch point from cluster 1. Alternatively, cluster 3 appeared to be a transition phase between the homeostatic cluster and either the motile or dystrophic clusters. These findings are similar to a previous report by Nguyen et al.^26^. Our analysis nominates genetic regulators of each cluster, and together these data can be used to guide further studies to test the plasticity or reversibility of microglia phenotype^53^. Furthermore, unlike other cell types that are terminally differentiated, it is plausible that microglia may transition in and out of transcriptomic states, underscoring the ‘snapshot’ nature of tissue omics.

We detected an AD-specific microglia phenotype, subcluster 1.5, within the homeostatic-marker-expressing cluster, cluster 1. The ‘homeostatic’ moniker is often invoked to describe a cluster expressing genes termed homeostatic because of their decreased expression upon microglial activation. Whether these genes truly have an active role in directing an activated or injured cell to resume a less reactive state or in maintaining a particular state is unknown. Nevertheless, we found that the cluster identified as ‘homeostatic’ using the typical homeostatic markers belies a complexity of microglia profiles that may hold clues to early or subtle microglial changes in response to AD pathology. As an example, subcluster 1.5 shows the highest expression of *P2RY12* as well as expression of genes that suggest another layer of activity. Of note, *P2RY12* is associated with active microglial phenotypes and is considered essential for the initial stages of motility during inflammation^54^. The most highly differentially regulated gene in subcluster 1.5 is *PDE4b*, a phosphodiesterase implicated in cognitive function^55^ and myeloid cell inflammatory activation^56,57^. PDE4b regulates levels of cAMP, which was shown to modulate microglia surveillance behavior^58^. Subcluster 1.5 also demonstrates enrichment of motility and calcium signaling pathways, which could suggest response to neuronal activity or extension of microglial processes^53^. Therefore, we speculate that subcluster 1.5 may represent early or subtle microglial phenotype changes in response to pathologic protein but not a fully immunologically activated phenotype.

As with all snRNA-seq studies, there are limitations to our study. Although PU.1 sorting provides a way to increase microglia nuclei for greater resolution of phenotypes at an individual subject level, it potentially selects for a specific population of microglia. Additionally, although known microglial and peripheral myeloid markers used to annotate cells increased confidence of a microglial designation, it is still possible that nuclei included may be a different brain myeloid cell type. DEGs that were identified using clusters defined by the same data are not necessarily properly controlled^59^; however, our dataset will allow others to use our clusters to mitigate this in the future. Although the total number of microglia from each subject is large, the total number of subjects studied is 22. Additional studies in larger cohorts are needed to validate these findings or perhaps give additional insight into microglial diversity. Gene expression is a useful molecular tool for cellular subtyping, but it does not always directly describe protein expression, localization or function^60^. Future studies assaying for a panel of proteins based upon the transcriptomic signatures reported here will be valuable for both validation as well as the spatial correlation with pathological features. Assessment of microglial phenotypes across brain regions can provide further context to the understanding of phenotypic heterogeneity. Another significant limitation is the use of autopsy brain tissue. It is possible that events just before death or postmortem changes contribute to the expression changes measured here. To mitigate the variability of tissue quality, we selected only tissues with a pH greater than 6.0 and a postmortem interval (PMI) less than 10 h.

In this study, we identified microglia states from isolated human postmortem brain nuclei. Among the nuclei meeting criteria for a microglia transcriptomic signature, an ‘aging signature’ was observed in all clusters in this study^23^, consistent with our older age cohort. Inflammaging^61^ may not only confound interpretations of gene expression profiles attributable to AD but may also contribute to the disease mechanisms hypothesized to drive AD. Additional studies exploring differences between younger controls and early-onset AD may also help to explore the aging, inflammaging and AD-specific signatures. Our identification of multiple internalization and trafficking clusters with varying metabolic and inflammatory gene expression patterns provides a platform for further studies. Finding an AD-specific subcluster within the population of microglia enriched for homeostatic gene expression also suggests that AD changes and, thus, molecular pathways driving AD can be identified in cells that have not yet been fully explored. The cluster-specific alterations in composition, gene expression and gene regulation in AD brain provide additional information to support tailored targeting of microglial physiological responses that will be critical moving forward in neurodegenerative disease therapeutics.

## Methods

### Human brain tissue

Our research complies with all relevant ethical regulations and was approved by the institutional review board at the University of Washington. Dorsolateral prefrontal cortex (dlPFC) tissue from human brains was obtained from the Neuropathology Core of the Alzheimer’s Disease Research Center at University of Washington after written informed consent. Patients (*n* = 12) were confirmed postmortem to have AD pathology (Alzheimer’s Disease Neuropathic Change (ADNC) score of 2–3; Table 1). Control individuals (*n* = 10) had low or no neuropathology postmortem (ADNC score 0–1; Table 1).

**Table 1:** Postmortem brain sample demographics

Group	Males	Females	Avg. age	Avg. PMI	ADNC	E2/3	E3/3	E3/4	E4/4
**Ctrl**	4	6	85.9	5.53	0–1	2	7	1	
**AD**	3	9	86.5	4.67	2–3		6	5	1
**Total**	7	15	86.23	5.42		2	13	6	1

AD, Alzheimer’s disease pathology; Avg. age, average years of age at death; *APOE* genotypes: *APOE* alleles 2/3 (E2/3), *APOE* alleles 3/3 (E3/3), *APOE* alleles 3/4 (E3/4) and *APOE* alleles 4/4 (E4/4); Ctrl, control; Avg. PMI, average postmortem interval in hours.

Brain samples were flash frozen and stored at −80°C. Criteria for inclusion included PMI ≤10 h, low comorbid pathology (Lewy bodies and hippocampal sclerosis) and a brain pH at autopsy ≥6.

### Isolation of nuclei for unsorted snRNA-seq

Nuclei from brain samples were isolated using protocols adapted from 10x Genomics Demonstrated Protocols and De Groot et al.^62^. In brief, four 2-mm punches of dlPFC gray matter were collected using a biopsy punch (Thermo Fisher Scientific) into a 1.5-ml microcentrifuge tube on dry ice. All buffer, solution and media recipes can be found in Supplementary Tables 1–7. Nuclei isolation used nuclei lysis buffer. The nuclei in nuclei suspension solution was layered onto 900 µl of percoll/myelin gradient buffer^62^. The gradient was centrifuged at 950*g* for 20 min at 4 °C with slow acceleration and no brake. Myelin and supernatant were aspirated, and the nuclei pellet was resuspended in resuspension buffer at a concentration of 1,000 nuclei per microliter and proceeded immediately to snRNA-seq.

### Isolation of nuclei for FANS

In brief, 100–250 mg of dlPFC gray matter was collected into a 1.5-ml microcentrifuge tube on dry ice. Brain tissue was homogenized as above. The homogenate was incubated at 4 °C under gentle agitation for 10 min, pelleted at 500*g* for 7 min at 4 °C and resuspended in 900 µl of percoll/myelin gradient buffer supplemented with protease and phosphatase inhibitors. The suspension was gently overlaid with 300 µl of nuclei suspension solution supplemented with protease and phosphatase inhibitors. The gradient was centrifuged at 950*g* for 20 min at 4 °C with slow acceleration and no brake. The myelin and supernatant were aspirated, and the nuclei pellet proceeded immediately to FANS.

### FANS

Nuclei were washed with cold FANS media (10% FBS, 10 mM HEPES, 100 μM ATA, 10% 10× HBSS, 0.5% Protector RNase Inhibitor, protease and phosphatase inhibitors and 1% saponin in nuclease-free water) and resuspended in FANS media at a concentration of 2–2.5 × 10^6^ nuclei per milliliter. Nuclei were incubated with 1% Human Fc Block (clone Fc1.3216, BD Biosciences) on ice for 10 min. Nuclei were labeled with either anti-PU.1-PE (clone 9G7, 1:50, Cell Signaling Technology) or IgG-PE isotype control (clone DA1E, 1:50, Cell Signaling Technology) for 4 h on ice, followed by three washes with cold FANS media and resuspended in FANS media supplemented with 10 μg ml^−1^ DAPI (Sigma-Aldrich). Nuclei were sorted using a FACSAria III (BD Biosciences) until 30,000 PU.1-positive nuclei were collected. Sorted nuclei were centrifuged at 1,000*g* for 10 min at 4 °C. The nuclei pellet was resuspended in resuspension buffer at a concentration of 1,000 nuclei per microliter and proceeded immediately to snRNA-seq.

### snRNA-seq

Single-nucleus libraries were generated using the Chromium Next GEM Single Cell 3′ GEM, Library and Gel Bead Kit version 3 (10x Genomics) according to the manufacturer’s protocol and a target capture of 10,000 nuclei. Gene expression libraries were sequenced on the NovaSeq 6000 platform (Illumina).

### Alignment and quality control

Gene counts were obtained by aligning reads to the hg38 genome (GRCh38-1.2.0) using CellRanger 3.0.2 software (10x Genomics). Reads mapping to precursor mRNA were included to account for unspliced nuclear transcripts. Most of our analysis was performed in R (R Development Core Team 2010). Droplets from 22 PU.1 sorted samples were combined using Seurat version 3.3 (ref. ^63^). Unsorted and PU.1 sorted droplets isolated from the same four subjects were combined using Seurat and analyzed in the same manner. Droplets containing fewer than 350 unique molecular identifiers, fewer than 350 genes or more than 1% mitochondrial genes were excluded from analysis. Ambient RNA was removed from the remaining droplets using SoupX^64^. Droplets containing multiple nuclei were scored using Scrublet^65^ and removed. In total, 200,948 nuclei with an average of 1,787 genes per nucleus remained in the dataset for further analysis.

### Normalization and cell clustering

Normalization and clustering of the nuclei were performed using Seurat version 3.3 (ref. ^63^). Data were normalized for read depth, and mitochondrial gene content was regressed out using Seurat’s SCTransform^66^. Individual sample variability was removed using Seurat’s Anchoring and Integration functions^63^. The top 5,000 variable genes were kept. Fifteen principal components (PCs) were used to create a shared nearest neighbors graph with *k* = 20. The modularity function was optimized using a resolution of 0.2 to determine clusters using the Louvain algorithm with multilevel refinement to determine broad cell types. Each cluster met a minimum threshold of 30 defining DEGs and comprised nuclei from more than 10% of the cohort (more than two individuals).

Clusters were annotated for cell type using manual evaluation for a set of known genetic markers^67^. A new Seurat object was made containing only the microglia nuclei (*n* = 127,371). Normalization, individual variability removal, integration and shared nearest neighbors graph creation were repeated as above on the microglia nuclei. Twenty PCs were chosen to account for a significant amount of the variance. Clusters were determined using the Leiden algorithm^68^ with method=igraph and weights=true. Clusters were highly conserved across analysis by Louvain, Louvain with multilevel refinement and Leiden algorithms. Homeostatic cluster subclustering for both the 22-sample dataset and the *APOE* ε3/ε3 allele dataset occurred after normalization, individual variability removal and integration as before and was performed using 10 PCs and the Louvain with multilevel refinement algorithm. Distribution of nuclei within each cluster was calculated using the ‘chisq.test’ function in R to compare the actual percentage of nuclei from either the AD or control group within the cluster to the expected proportion of nuclei that would be contributed based on dataset composition. *P* values from the chi-squared tests were adjusted using false discovery rate (FDR) and considered significant if adjusted *P* < 0.05.

### snRNA-seq differential gene expression and GSEA

Differential gene expression analysis of the clusters was performed with the MAST algorithm. Genes tested had expression in at least 25% of the nuclei in the cluster. DEGs had an FDR-adjusted *P* < 0.05 and a log fold change > 1.25. Cluster 1 was annotated as inactivated, often referred to as ‘homeostatic’ in single-nucleus studies of microglia. Differential gene expression analysis was repeated as above comparing each other cluster to cluster 1. GSEA was performed in ClusterProfiler^69^ modified to use a set seed for reproducibility, using the GO, KEGG and Reactome pathway sets, version 7.2. Enriched pathways had an FDR-adjusted *P* < 0.05. We considered pathways to be representative if significant results included similar genes and biological functions in at least two of the three major databases (GO, KEGG and Reactome).

### GO biological process clustering

A complementary approach to GSEA is to perform biological process ontology clustering to identify a more extensive set of terms associated with the gene list. To further characterize the ELN clusters (3, 5 and 6), we implemented this approach to get a more refined examination of the biologically linked process driven by each cluster. We employed several different approaches to perform this analysis, ultimately choosing the Cytoscape network clustering application ClueGO^70,71^, which has been employed extensively in recent years for this purpose^72–75^. This creates a network of genetically linked processes that goes beyond a singular GO term hit, providing greater confidence that a basic biological process is being impacted based on the differential expression of a particular set of genes. Clusters 3, 5 and 6 were each independently submitted for analysis using a one-sided positive enrichment algorithm that employs hypergeometric testing, with a kappa threshold of 0.4 to optimize the biological process connections. Each term drawn into the network was initially filtered for multiple testing corrections threshold of *P* < 0.05 and hierarchically weighted for terms with a Benjamini–Hochberg correction value of *P* < 0.01. As this procedure is performed for validation and visualization, we trimmed networks of lesser-ranked terms to permit ease of visualization.

### Trajectory and lineage analysis

Trajectory analysis was performed using Monocle3 (ref. ^45^) on multiple permutations of our downsampled dataset. The data were downsampled to 1,000, 2,000, 3,000 or 5,000 nuclei per cluster and transferred to a cell dataset object, and Monocle3 ‘learn_graph’ was run. Principal component analysis and uniform manifold approximation and projection (UMAP) embeddings were extracted from the Seurat object. We applied the algorithm both with and without a defined starting point. The 5,000 nuclei-per-cluster downsampled data began to break down the ability of Monocle to form a consistent trajectory, whereas the 1,000, 2,000 and 3,000 multiple permutations consistently formed something similar to the representative image in Fig. 5 (3,000 nuclei per cluster).

### Gene regulatory network inference

Regulons were inferred using the SCENIC workflow in Python (pySCENIC)^76,77^. We randomly selected 2,000, 3,000 or 5,000 nuclei in each cluster (or, if they have fewer than this number, all the nuclei in the cluster) to reduce the computational time and have proportional representation of all the clusters. We made five subsets of each combination and repeated the analysis twice for each subset to assess the consistency of the regulons in the analysis. First, we used normalized counts with highly variable genes to generate the co-expressing regulatory network modules using the machine learning algorithm GRNBoost2 with function ‘grn’ and default settings^76^. Second, the modules were filtered using the ‘-ctx’ function, which uses *cis*-regulatory motif analysis (RcisTarget) to keep only modules enriched for putative target genes of the transcription factor. Regulons are identified by combining multiple modules for a single transcription factor. Third, the AUCell function was used to calculate the regulon activity for each nucleus. Regulon specificity scores were calculated for each regulon in every cluster. Ranking specificity scores identified the top 10 regulons for a specific cluster for a given subset of the dataset and the consistency of the findings across subsets.

### Immunostaining of human tissue

Dissected tissues from the dlPFC of the 22 cases in the cohort were fixed with paraformaldehyde and paraffin embedded. Samples were sectioned at 15 µm and deparaffinized before immunostaining. Slides were boiled in citrate buffer (Sigma-Aldrich, C9999) for 20 min and then transferred into blocking buffer (10% donkey serum, 0.1% Trition X-100 and 0.05% Tween 20 in TBS) for 1 h at room temperature. Slides were incubated in primary antibodies (anti-LAMP1 1:100, Invitrogen, 14-1079-80; anti-Iba-1 1:250, Abcam, ab5076; anti-dsDNA 1:250, Millipore, MAB1293; anti-PTGDS/PGD2 1:100, R&D Systems, MAB10099; anti-P2RX7 1:100, Santa Cruz Biotechnology, sc-514962; anti-P2RY12 1:50, Alomone, APR-012; and anti-PDE4B 1:50, LSBio, LS-C173292-100) overnight at 4 °C. Slides were rinsed three times in TBS-T for 5 min before secondary antibody (Thermo Fisher Scientific, Alexa Fluor 488 donkey anti-goat, A11055; Thermo Fisher Scientific, Alexa Fluor 555 donkey anti-mouse, A31570; Alexa Fluor 555 donkey anti-rabbit 555, A31572; Alexa Fluor 647 donkey anti-mouse, A31571; or Alexa Fluor 647 donkey anti-rabbit, A32795) incubation for 1 h at room temperature. Slides were then stained with DAPI (1:1,000, Millipore, D8417) for 5 min, followed by three 5-min TBS-T washes. True Black (Thermo Fisher Scientific, NC1125051) diluted 1:20 in 70% ethanol was added to the slides for 50 s, followed by two additional 5-min washes in TBS before being mounted with Fluoromount-G (Southern Biotech, 0100-01). Slides were imaged using either an Olympus FluoView-1000 confocal microscope or a spinning disk confocal microscope (Nikon A1R with Yokogawa W1 spinning disk head) with ×40 and ×100 oil objectives, and the maximum projection of *z*-stack images was generated. Images were despeckled using Fiji.

### Statistics and reproducibility

No statistical methods were used to pre-determine sample sizes, but our sample sizes are similar to those reported in previous publications^22,25,26^. Data collection and analyses were not performed blinded to the conditions of the experiments. No data were excluded from the analyses. The main snRNA-seq dataset of PU.1 enrichment from dlPFC is from 22 humans (12 AD and 10 healthy aged donors), all aged >60 years. Because the samples were obtained from humans, and the study was designed to detect differences between AD cases and healthy aged brains, the samples were not randomized between conditions. We counterbalanced sequencing batches by sex and disease status such that all conditions were present in each sequencing batch. Statistical analysis for pseudobulk RNA-seq data and snRNA-seq data used DESeq2 and MAST, respectively. DESeq2 is designed to model the RNA-seq count data by a negative binomial distribution, and MAST is designed to model the zero inflation observed in snRNA-seq data. For statistical analysis of cluster proportion, counts data were used, which does not rely on a normal distribution. When the dataset was downsampled for trajectory analysis and regulon detection, the nuclei were randomly downsampled to generate multiple iterations of the dataset that evenly represented the clusters and kept the samples in their original proportions. For trajectory analysis, three downsampled permutations at three downsample resolutions (1,000, 2,000 or 3,000 nuclei per cluster) were analyzed for a total of nine iterations, resulting in similar findings as those displayed in Fig. 4. For pySCENIC regulon detection, the dataset was downsampled with five permutations at 1,000 nuclei per cluster and three permutations at 2,000 and 3,000 nuclei per cluster. Each of these permutations was run through the pySCENIC pipeline multiple times, resulting in 27 instances of regulon detection. These 27 instances were compiled into the consistency scores displayed in Fig. 3 and Extended Data Fig. 6. Experiments involving immunohistochemistry of human tissue were replicated on, at minimum, samples from five humans, and images displayed are representative of staining observed in multiple fields of at least three human samples.

### Reporting Summary

Further information on research design is available in the Nature Portfolio Reporting Summary linked to this article.

## Supplementary information

Supplementary Information Supplementary Tables 1–7.

Reporting Summary

Supplementary Data 1 Supplemental data for the 22-sample Mixed *APOE* genotype dataset, including DEGs compared to all other clusters and compared to the HM cluster, pathways for each cluster identified by GSEA and pseudobulk results comparing AD to control cases within each cluster.

Supplementary Data 2 Supplemental data for the 13-sample *APOE* ε3/ε3 dataset, including DEGs compared to all other clusters and compared to the HM cluster, pathways for each cluster identified by GSEA, percentage of overlap of DEGs with the 22-sample Mixed *APOE* genotype dataset clusters and pseudobulk results comparing AD to control cases within each cluster.

## Data Availability

The entire anonymous dataset generated for this resource in its raw and Seurat object processed forms is available via Synapse (https://www.synapse.org/#!Synapse:syn51272688). The data are available under controlled use conditions set by human privacy regulations. To access the data, a data use agreement is needed. This registration is in place solely to ensure anonymity of the study participants. All other study data are available from the corresponding author upon reasonable request. This resource also used the publicly available human hg38 genome (GRCh38-1.2.0).

## Code availability

The scripts used to generate our analyses are available at https://github.com/keprater/jayadevlab_pu.1_project. The container needed to run the scripts with the same Seurat and other package versions as are used in the code is available for download at https://hub.docker.com/r/keprater/jayadevlab/tags or in the Synapse project with the dataset. Use container Tag 6.3 to replicate our analyses with the same package versions.
