# Supplementary material for: Human microglia show unique transcriptional changes in Alzheimer’s disease
Source: Nat Aging. 2023 May 29;3(7):894–907. doi: 10.1038/s43587-023-00424-y (PMC10353942; doi:10.1038/s43587-023-00424-y)
Supplement: Supplementary file 1 — Supplementary Tables 1–7. [file 43587_2023_424_MOESM1_ESM.pdf]

nature aging

Resource

https://doi.org/10.1038/s43587-023-00424-y

Human microglia show unique transcriptional changes in Alzheimer's disease

In the format provided by the authors and unedited

Prater, Green, et al. Supplemental Tables

Supplemental Table 1: Complete demographic information on the cohort

Sample	Study Designation	Coded AGE	SEX	Race	APOE Genotype	PMI	ADNC Score
1	AD	90+	F	White	3,3	5.08	3
2	AD	74	F	White	3,4	4.42	3
3	AD	90+	F	Mixed	3,3	5.08	2
4	AD	90+	F	White	3,3	3.75	3
5	AD	60	F	Unknown/Unreported	3,3	5.75	3
6	AD	86	F	White	3,3	8.07	2
7	AD	87	F	White	3,4	4.87	3
8	AD	90+	F	White	3,4	4.25	3
9	AD	77	F	White	4,4	3.33	3
10	AD		M	White	3,3	3.33	3
11	AD	83	M	White	3,4	3	3
12	AD	90+	M	White	3,4	4.58	3
13	Ctrl	90+	F	Hispanic / Latino	2,3	4.33	0
14	Ctrl	90+	F	White	3,3	3.75	1
15	Ctrl	90+	F	White	3,3	6.97	0
16	Ctrl	80	F	White	3,3	8.13	0
17	Ctrl	90+	F	White	3,3	7.72	1
18	Ctrl	70	F	White	3,4	7	1
19	Ctrl	74	M	White	2,3	4.83	0
20	Ctrl	84	M	White	3,3	3.92	1
21	Ctrl	90+	M	White	3,3	8.17	1
22	Ctrl	82	M	White	3,3	7.75	1

Ctrl = Control, AD = Alzheimer's Disease pathology, Coded Age = age at death in years. Ages greater than 90 are coded 90+ to maintain anonymity, F = Female, M = Male, Race = self-reported race, *APOE* Genotypes: *APOE* alleles ε2/ε3 (2/3), *APOE* alleles ε3/ε3 (3/3), *APOE* alleles ε3/ε4 (3/4), or *APOE* alleles ε4/ε4 (4/4), PMI = post-mortem interval in hours, ADNC = Alzheimer's Disease Neuropathic Change

Recipes for Nuclei Extraction and FANS Buffers:

Supplemental Table 2: Myelin gradient buffer (Store at 4°C)

**Reagent**	**Volume for 1 Liter**
$NaH_2PO_4 \cdot H_2O$ (Fisher Scientific, S369-500), adjust to pH 7.4 with 3.56g/L $Na_2HPO_4 \cdot H_2O$ (Fluka, 71643)	0.78 g
NaCl (Fisher Scientific, S271-3)	8.0 g
KCl (Fisher Scientific, P217-500)	0.4 g
Glucose (Sigma, G7021-1KG)	2.0 g
BSA (VWR, EM-2930)	0.2%

Supplemental Table 3: Nuclei buffer (NB) (Store at 4°C)

**Reagent**	**Volume for 10mL**
Nuclease-free water into a 15 ml conical tube. (Fisher Scientific, M46000)	9.85 mL
1 M Tris-HCl, pH 7.5 (ThermoFisher, 15567027)	100 µL
5 M NaCl (ThermoFisher AM9760G)	20 µL
1 M MgCl2 (ThermoFisher Scientific, AM9530G)	30 µL

Supplemental Table 4: Nuclei lysis buffer (NLB) (make same-day)

**Reagent**	**Volume for 1 Sample**
Nuclei Buffer	727 µL
10% NP-40 alternative (final concentration 0.1%)	10 µL
Protease inhibitors in DPBS (Sigma-Aldrich, 4693124001)	142.9 µL
1mM ATA in NB (make fresh evening prior)	112.5 µL
PMSF (Tocris Bioscience, 4486)	10 µL
Phosphatase inhibitors (Sigma-Aldrich, P5726-1ML)	5 µL
Protector RNase inhibitor (final concentration 1 U/µl). (Sigma, 3335402001)	28.25 µL
**Total Volume**	1,035.5 µL

Supplemental Table 5: Nuclei suspension solution (NSS) (make same-day)

**Reagent**	**Volume for 1 Sample**
DPBS (Sigma-Aldrich, D8537-500ML)	637 µL
10% BSA (final concentration 1%) (Sigma-Aldrich, A1595-50mL)	100 µL
1mM ATA in DPBS (make fresh evening prior)	100 µL
7x Protease inhibitors in DPBS (Sigma-Aldrich, 4693124001)	142.9 µL
1M PMSF (Tocris Bioscience, 4486)	10 µL
Protector RNase inhibitor (final concentration 1.0 U/µl) (Sigma, 3335402001)	5 µL
Phosphatase inhibitors (Sigma-Aldrich, P5726-1ML)	5 µL
**Total Volume**	1 mL

Supplemental Table 6: Percoll/myelin gradient buffer solution (PMB) (make same day)

**Reagent**	**Volume for 1 Sample**
Myelin gradient buffer	412 μL
10x HBSS (Fisher Sci., 14185052)	30 μL
1mM ATA in myelin gradient buffer (make fresh evening prior)	100 uL
1.5M NaCl	25 μL
Percoll (Fisher Sci., 17-089-101) (Do not use if crystals have precipitated out)	270 μL
Protector RNase Inhibitor (final concentration 1.0 U/μl) (Sigma Aldrich, 3335402001)	5 μL
Phosphatase inhibitors (Sigma-Aldrich, P5726-1ML)	5 μL
7x Protease inhibitors in myelin gradient buffer (Sigma-Aldrich, 4693124001)	142.9 μL
PMSF (Tocris Bioscience, 4486)	10 μL
**Total Volume**	1 mL

Supplemental Table 7: FACS Media (FM) (store at 4°C)

**Reagent**	**Volume for 1 sample**
Nuclease free H_2O (Fisher Scientific, M46000)	7.7 mL
HEPES (Invitrogen, 15630080)	100 uL
10x HBSS without Mg/Ca (Fisher Scientific, 14185052)	1 mL
FBS 10%	1 mL
Protease inhibitors (Sigma-Aldrich, 4693124001) (dissolve tablet in 9.8mL of the above media at room temp and chill on ice before adding the solutions below)	"~200 uL"
PMSF (Tocris Bioscience, 4486)	100 uL
Phosphatase inhibitors (Sigma-Aldrich, P5726-1ML)	50 uL
Protector RNase Inhibitor (Sigma Aldrich, 3335402001)	50 uL
100 uM ATA in dPBS (Sigma-Aldrich, A1895) good at -20C for 1 month	1 mL
Total Volume	11.2 mL
