# Supplementary material for: Human microglia show unique transcriptional changes in Alzheimer’s disease
Source: Nat Aging. 2023 May 29;3(7):894–907. doi: 10.1038/s43587-023-00424-y (PMC10353942; doi:10.1038/s43587-023-00424-y)
Supplement: Supplementary file 2 — Reporting Summary [file 43587_2023_424_MOESM2_ESM.pdf]

nature portfolio

Corresponding author(s): Suman Jayadev

Last updated by author(s): Mar 31, 2023

Reporting Summary

Nature Portfolio wishes to improve the reproducibility of the work that we publish. This form provides structure for consistency and transparency in reporting. For further information on Nature Portfolio policies, see our Editorial Policies and the Editorial Policy Checklist.

Statistics

For all statistical analyses, confirm that the following items are present in the figure legend, table legend, main text, or Methods section.

n/a	Confirmed	
☐	☒	The exact sample size (*n*) for each experimental group/condition, given as a discrete number and unit of measurement
☐	☒	A statement on whether measurements were taken from distinct samples or whether the same sample was measured repeatedly
☐	☒	The statistical test(s) used AND whether they are one- or two-sided<br>*Only common tests should be described solely by name; describe more complex techniques in the Methods section.*
☐	☒	A description of all covariates tested
☐	☒	A description of any assumptions or corrections, such as tests of normality and adjustment for multiple comparisons
☒	☐	A full description of the statistical parameters including central tendency (e.g. means) or other basic estimates (e.g. regression coefficient) AND variation (e.g. standard deviation) or associated estimates of uncertainty (e.g. confidence intervals)
☐	☒	For null hypothesis testing, the test statistic (e.g. *F*, *t*, *r*) with confidence intervals, effect sizes, degrees of freedom and *P* value noted<br>*Give P values as exact values whenever suitable.*
☒	☐	For Bayesian analysis, information on the choice of priors and Markov chain Monte Carlo settings
☒	☐	For hierarchical and complex designs, identification of the appropriate level for tests and full reporting of outcomes
☒	☐	Estimates of effect sizes (e.g. Cohen's *d*, Pearson's *r*), indicating how they were calculated

Our web collection on statistics for biologists contains articles on many of the points above.

Software and code

Policy information about availability of computer code

Data collection	No unique software was used for data collection.
Data analysis	Scripts, and the container (Tag 6.3) needed to run them with the appropriate versions of the packages listed, are provided via GitHub and DockerHub at https://github.com/keprater/jayadevlab_pu.1_project and https://hub.docker.com/r/keprater/jayadevlab/tags. CellRanger 3.0.2 with GRCh38-1.2.0, R version (v)4.0.0, Seurat v3.1.5, SoupX v1.4.5, Scrublet 0.2.1, clusterProfiler v3.16.1, Monocle3 v0.2.2, pySCENIC v0.11.2, ggplot2 v3.3.1, Matrix v 1.2-18, Reticulate v1.16, Leiden v0.3.3, sctransform v0.2.1, SingleCellExperiment v1.10.1, GenomeInfoDb v1.24.2, Numpy v1.20.0, Pandas v1.3.3, Matplotlib v3.4.3, and Python v3.8.10 were utilized.

For manuscripts utilizing custom algorithms or software that are central to the research but not yet described in published literature, software must be made available to editors and reviewers. We strongly encourage code deposition in a community repository (e.g. GitHub). See the Nature Portfolio guidelines for submitting code & software for further information.

Data

Policy information about availability of data

All manuscripts must include a data availability statement. This statement should provide the following information, where applicable:

- Accession codes, unique identifiers, or web links for publicly available datasets
- A description of any restrictions on data availability
- For clinical datasets or third party data, please ensure that the statement adheres to our policy

The entire anonymous dataset generated for this resource in its raw and Seurat object processed form is available via Synapse (https://www.synapse.org/#!Synapse:syn51272688). The data are available under controlled use conditions set by human privacy regulations. To access the data, a data use agreement is needed. This registration is in place solely to ensure anonymity of the study participants. All other study data are available from the corresponding author upon reasonable request. This resource also used the publicly available human hg38 genome (GRCh38-1.2.0).

Human research participants

Policy information about studies involving human research participants and Sex and Gender in Research.

Reporting on sex and gender	Our dataset contains 7 males and 15 females based on self-reported sex. We did not attempt sex-based analyses because of the small number of males in the study. Gender information was not collected consistently for these participants, so we do not report it.
Population characteristics	Our dataset contains sequencing data from the post-mortem brains of 22 individuals all over the age of 60 with an average age of 86.2 years. 10 individuals (6 female, 4 male) are considered controls, with an Alzheimer's Disease Neuropathic Change (ADNC) score of 0-1, and an NIA-AA score of not-AD to Low. 12 individuals (9 female, 3 male) are considered to have Alzheimer's Disease pathology, with an ADNC score of 2-3, and an NIA-AA score of Intermediate to High. 13 of those individuals (7 control and 6 AD) were APOE e3/e3 genotype. 6 individuals (5 AD and 1 control) were APOE e3/e4 genotype. One AD individual was APOE e4/e4 genotype. Two controls were APOE e2/e3 genotype. Please see Supplemental Table 1 for additional population characteristic information.
Recruitment	Participants were recruited to autopsy-based studies through either the ACT study or the UW ADRC. The participants are primarily white, and female. This bias is a reflection of the demographics of the Seattle area, and therefore the cohorts of the ACT and ADRC studies. Otherwise, selection bias is limited to those individuals willing to donate their brain.
Ethics oversight	Protocols for ACT and studies within the ADRC were approved by the IRB at the University of Washington. Our study is considered exempt since it uses post-mortem and anonymous participant information.

Note that full information on the approval of the study protocol must also be provided in the manuscript.

Field-specific reporting

Please select the one below that is the best fit for your research. If you are not sure, read the appropriate sections before making your selection.

☒ Life sciences ☐ Behavioural & social sciences ☐ Ecological, evolutionary & environmental sciences

For a reference copy of the document with all sections, see nature.com/documents/nr-reporting-summary-flat.pdf

Life sciences study design

All studies must disclose on these points even when the disclosure is negative.

Sample size	Our dataset contains 22 brain samples of dorsolateral prefrontal cortex from 10 control and 12 Alzheimer's Disease pathology individuals. These individuals were all over the age of 60, and there were 7 male and 15 female samples. No statistical methods were used to pre-determine sample sizes but our sample sizes are similar to those reported in previous publications. In this study, we also enriched our dataset for PU.1, a myeloid marker. This allowed us to sequence and analyze the largest microglia/sample dataset to-date. While a larger sample size of individuals will always be more informative, this dataset provides the greatest depth of microglia sequencing so far.
Data exclusions	No data was excluded from analysis due to statistical rationale. Data from cell types other than microglia were excluded from analysis and interpretation in this study since we enriched for PU.1 and therefore may not have a representative population of other cell types.
Replication	The pySCENIC regulon detection and Monocle3 trajectory analysis were replicated multiple times (27 for regulons, 9 for trajectory) across multiple permutations of the downsampled dataset to generate the cosnistent findings displayed in the figures. Findings from the single-nucleus RNAseq gene expression analyses were replicated in human brain tissue utilizing immunohistochemistry. Immunohistochemistry was replicated on 5-22 human samples and images are representative of staining observed in multiple fields across at least three individual humans. We also confirmed that the major findings of the snRNASeq dataset replicated similarly in our subset cohort of all APOE e3/e3 individuals. While not a separate dataset replication, this finding is important for future studies.

Randomization	Samples were allocated to experimental groups based on their pathological characterization by the UW Neuropathology Core. Sex and Age were matched as well as possible across the experimental groups and during sequencing batches.
Blinding	Investigators were not blinded to study grouping during analysis of the dataset since that variable was relevant to the statistical analysis of the data.

Reporting for specific materials, systems and methods

We require information from authors about some types of materials, experimental systems and methods used in many studies. Here, indicate whether each material, system or method listed is relevant to your study. If you are not sure if a list item applies to your research, read the appropriate section before selecting a response.

Materials & experimental systems

n/a	Involved in the study
☐	☒ Antibodies
☒	☐ Eukaryotic cell lines
☒	☐ Palaeontology and archaeology
☒	☐ Animals and other organisms
☒	☐ Clinical data
☒	☐ Dual use research of concern

Methods

n/a	Involved in the study
☒	☐ ChIP-seq
☐	☒ Flow cytometry
☒	☐ MRI-based neuroimaging

Antibodies

Antibodies used	Primary antibodies: Anti-LAMP1 1:100 Invitrogen CAT#14-1079-80; anti-Iba-1 1:250 Abcam CAT#ab5076; anti-dsDNA 1:250 Millipore CAT#MAB1293; anti-PTDGS/PGD2 R&D Systems CAT#MAB10099 1:100; anti-P2RX7 Santa Cruz CAT#sc-514962 1:100; anti-P2RY12 Alomone CAT#APR-012 1:50; anti-PDE4B LSBio CAT#LS-C173292-100 1:50<br><br>Secondary antibodies: All secondary antibodies were used at 1:500. Thermofisher Alexa Fluor 488 Donkey anti-Goat CAT# A11055; Thermofisher Alexa Fluor 555 Donkey anti-Mouse CAT#A31570; Alexa Fluor 555 Donkey anti-Rabbit 555 CAT#A31572; Alexa Fluor 647 Donkey anti-Mouse CAT#A31571; or Alexa Fluor 647 Donkey anti-Rabbit CAT#A32795
Validation	All primary antibodies purchased and used for this study were validated by the manufacturer for use in human tissue for IHC-P.

Flow Cytometry

Plots

Confirm that:

☒ The axis labels state the marker and fluorochrome used (e.g. CD4-FITC).

☒ The axis scales are clearly visible. Include numbers along axes only for bottom left plot of group (a 'group' is an analysis of identical markers).

☒ All plots are contour plots with outliers or pseudocolor plots.

☒ A numerical value for number of cells or percentage (with statistics) is provided.

Methodology

Sample preparation	Human flash frozen post-mortem brain tissue from the dorsolateral prefrontal cortex was lysed and nuclei isolated utilizing a percoll gradient. PU.1 or the appropriate isotype control were added to the sample for four hours on ice prior to sorting. DAPI was added 5 minutes prior to the sort time.
Instrument	BD FACS Aria III
Software	BD FACS Diva software provided with the cytometer. No custom software or code was used.
Cell population abundance	The PU.1 positive population selected within the sort gate was approximately 10% of the total DAPI positive nuclei population for any given sample. The purity of the population was not 100% as the PU.1 staining does not generate a population that completely separates from the background nuclei. We later removed cell types that were not microglia from our dataset during analysis post-sequencing.
Gating strategy	Since these are nuclei, the primary gating strategy utilized DAPI rather than the FSC/SSC to select a starting population from which to detect staining. The DAPI height and DAPI area were utilized to detect the different DAPI positive populations. The gate was centered around the smallest DAPI positive population, though did include a portion of the second DAPI population. The PU.1 population was identified by it's higher PE staining versus autofluorescence detected by the FITC channel. The main population of nuclei creates a diagonal, and while the isotype control does not demonstrate PE staining outside of that

diagonal, the PU.1 antibody generates a population that moves away from the main diagonal of the nuclei. These are illustrated in Supplemental Figure 1A/B.

☒ Tick this box to confirm that a figure exemplifying the gating strategy is provided in the Supplementary Information.
